# Beyond probes and charts: The AI revolution reshaping diagnosis, prognosis, and treatment decision-making in periodontology

**DOI:** 10.34172/japid.025.4144

**Published:** 2025-11-16

**Authors:** Mohammadreza Talebi Ardakani, Sajad Jahantigh

**Affiliations:** Department of Periodontics, School of Dentistry, Shahid Beheshti University of Medical Sciences, Tehran, Iran

## To the Editor,

 Artificial intelligence (AI) is a relatively new scientific discipline whose development can be traced back to its inception in 1956. During the planning of the Handbook of AI, researchers sought to comprehensively describe the expansion of this field, acknowledging its continuous evolution as methodologies and areas of focus have changed over time. The term “AI” refers to machines performing tasks typically associated with human capabilities. Within AI, machine learning (ML) focuses on algorithms that identify and internalize statistical patterns within data, enabling predictions for previously unseen data. A specialized form of ML, deep learning, uses multiple computational layers to process, learn from, and infer information from complex datasets, such as images and other high-dimensional data.^1,2^

 Periodontitis is a chronic, multifactorial inflammatory disease caused by dysbiotic dental plaque biofilms, leading to the progressive destruction of the tooth-supporting apparatus. Clinically, the disease manifests as loss of periodontal attachment and alveolar bone, as evidenced by clinical attachment loss and radiographic assessment, accompanied by periodontal pocketing and gingival bleeding. As one of the most prevalent oral diseases worldwide, its burden extends beyond dental complications: periodontitis results in significant tooth loss, functional impairment, and aesthetic deficiencies, contributing to social inequality and reduced quality of life.^3^ The widespread prevalence and economic impact associated with periodontal care emphasize the necessity for a transformative approach, one that is now emerging through data-driven and AI-based precision periodontology.

 Traditional periodontal diagnostics depend heavily on operator skill, intra-examiner consistency, and subjective interpretation of bone levels, pocket depth, and clinical attachment loss. Variability across clinicians and imaging standards has historically constrained reproducibility.

 A comprehensive scoping review identified over 6,000 studies exploring AI-based approaches to periodontal diagnostics and decision-making. Most investigations focused on deep learning, particularly convolutional neural networks (CNNs), applied to radiographic and intraoral image analysis. These models consistently achieved diagnostic accuracy, sensitivity, and specificity comparable to or exceeding that of experienced clinicians in detecting and classifying alveolar bone loss, intrabony defects, furcation involvement, gingivitis, biofilm accumulation, and calculus. Ensemble frameworks and task-oriented CNN architectures further enhanced reliability, reproducibility, and diagnostic speed. Collectively, the evidence highlights AI’s capacity to transform periodontal care through precision-driven assessment and individualized treatment planning.^3^

 Parallel evidence from systematic and scoping reviews confirms that AI, especially when integrated with advanced imaging modalities such as two-dimensional radiography and cone-beam computed tomography, delivers highly accurate detection and staging of periodontitis. CNNs and hybrid deep learning computer-aided diagnosis (CAD) models demonstrated high diagnostic accuracy (73%–98.6%) for detecting and staging periodontitis, often performing at or near expert clinician levels. These systems proved especially effective in detecting radiographic bone loss and benefited further from integration with clinical variables, such as probing depth and attachment level. Supervised ML systems employing diverse predictive variables repeatedly outperform traditional risk assessment and statistical models, with neural networks and multi-algorithm configurations proving particularly effective for predicting tooth-related outcomes and disease progression. The integration of AI technologies into periodontal practice represents a significant step toward standardized, efficient, and patient-centered care.^4-6^

 A cohort study of 7,840 periodontitis patients demonstrated that ML using the RuleFit algorithm effectively identifies key predictors of tooth loss, including age, periodontal diagnosis, baseline tooth loss, furcation involvement, and mobility. The models exhibited strong performance (AUROC = 0.71; AUPRC = 0.66) and improved interpretability compared to traditional regression, underscoring the promise of explainable AI for accurate risk stratification and personalized, data-driven periodontal care.^7^

 Collectively, these findings affirm that AI-driven technologies are reshaping periodontal screening, diagnosis, prognosis, and outcome prediction through precision-driven analysis and individualized treatment planning. Despite ongoing limitations such as data heterogeneity, sample imbalance, and variable methodological quality, the integration of AI represents a decisive step toward standardized, efficient, and patient-centered periodontal care, defining the emerging paradigm of data-driven, algorithm-assisted precision periodontology.^3-7^

 Despite its promise, AI introduces several ethical questions for periodontology. Algorithmic transparency, data privacy, and bias constitute critical points of attention. Many training datasets originate from limited patient populations, risking inequitable model generalization. Explainable AI frameworks should thus accompany all clinical decision-support tools to ensure interpretability and accountability.

 Data standardization also demands interdisciplinary collaboration between periodontists, computer scientists, and biomedical engineers. Education will play a crucial role: future clinicians must be literate not only in root morphology and pocket depth, but also in data flow, model validation, and algorithmic bias. The field will evolve from manual probing to digital reasoning, a transition requiring deliberate ethical and educational preparation.^8-10^

 The trajectory of periodontology is converging toward integrative precision workflows, in which clinical data, imaging, and biological signals seamlessly interconnect through AI-enabled platforms. Emerging multicenter repositories and interoperable data infrastructures will generate the volume and diversity required for robust algorithmic training, ensuring reproducibility and clinical reliability. Conventional periodontal charts are evolving beyond static documentation into dynamic intelligence hubs capable of continuous learning, model refinement, and longitudinal patient monitoring.

 This transformation envisions a clinical ecosystem where AI systems autonomously flag susceptibility profiles, stratify risk, and recommend individualized therapeutic timelines. Predictive interfaces may soon estimate the likelihood of tissue regeneration following surgical therapy or anticipate disease recurrence under maintenance care. Such capabilities, once speculative, now represent an imminent stage in the maturation of evidence-based and data-driven periodontology.

 Yet, amid technological optimism, the profession must reaffirm the central role of human clinical insight. Despite its analytical supremacy, AI remains incapable of replicating empathy, moral judgment, or the interpretive understanding of patient-specific context. The clinician’s expertise functions as the pivotal mediator transforming algorithmic output into therapeutic reasoning. Within this renewed perspective, AI and periodontology should not stand as opposing forces, but rather as complementary partners where algorithmic precision is shaped, verified, and humanized by clinical judgment. Thus, AI is no longer merely a diagnostic aid; it is actively redefining the entire spectrum of periodontal care from early detection and classification to personalized prognosis, clinical decision-making, and outcome prediction. The discipline’s future will be profoundly data-driven, integratively intelligent, and ethically informed. As AI replaces mechanical probing and paper charting with digital reasoning and real-time analytics, it introduces a new instrument of clinical truth: the algorithm.

 What remains constant, however, is the clinician’s responsibility to merge innovation with empathy, ensuring that as intelligence becomes artificial, care remains profoundly human.

## Competing Interests

 The authors declare that they have no competing interests regarding Authorship and/or publications of this paper.
